# Power Maximisation of Wind Energy Using Wind Speed Sensors on Stewart Island

**DOI:** 10.3390/s22218428

**Published:** 2022-11-02

**Authors:** Navid Majdi Nasab, Jeff Kilby, Leila Bakhtiaryfard

**Affiliations:** 1 Electrical and Electronic Engineering Department, School of Engineering, Computing and Mathematical Sciences Auckland University of Technology, Auckland 1010, New Zealand; 2 Technology Research Department, R&D Center, Fusheng Industrial Co., Ltd., Taipei 24158, Taiwan

**Keywords:** wind direction, sensor, power, WR PLOT view, Homer Pro

## Abstract

This paper evaluates the feasibility of using wind power for power supply to coastal communities isolated from the main supply grid. The case study is Stewart Island, where the cost of electricity provided by a central diesel power station is higher than the grid network in New Zealand. The Princeton Ocean Model (POM) conducted by MetOcean Solutions Limited (MSL) is used to find Foveaux as an optimized site for generating wind power. Global Wind Atlas is used to plot the wind rose of current wind patterns in New Zealand. In the next step, wind speed data from each site are imported from the NASA database to WRPLOT view software and Homer Pro to find wind frequency distribution and output power in the area. The maximum annual power can be seen in WSW (32,299 kW hours/year), SW (20,111 kW hours/year) and W (15,622 kW hour/year) directions, respectively.

## 1. Introduction

The World Energy Outlook 2019 clarifies the effect of recent decisions on energy systems in the future. If no policy actions are taken, then the current trend of energy demand is anticipated to increase by 1.30% per year until 2040, leading to an increase in emissions [1]. Nowadays, with the increasing global energy demand and global climate change, two essential solutions have been considered to tackle this issue: (i) reducing the cost of energy and (ii) finding new sources of energy that are environment friendly [2]. With the negative climate impact of fossil fuel power generation and the requirement of global policy to shift toward a green mix of energy production, investment in renewable energy is an opportunity in developing countries. However, a poor economy associated with limited income, funds availability and regulations governing project funding and development are key factors that challenge investors in the energy sector [3].

The New Zealand government has set the country’s goal to reduce greenhouse gas emissions by 30% from 2005 levels by 2030 and 80% by 2050 [4]. Between 1990 and 2019, Green House Gas (GHG) emissions increased from 41,114 kilo tonnes of carbon dioxide equivalent (kt CO_2_-e) to 82,318 kt CO_2_-e [5]. The New Zealand Government ratified the 2015 Paris Accord on 4 October 2016 and, as a result, Intended Nationally Determined Contribution (INDC) became a Nationally Determined Contribution (NDC). NDC target is to reduce GHG emissions to 30% below the 2005 level (83.7 Mt CO2-e) by 2030 (58.6 Mt) and net zero by 2050 [6,7]. However, this is a net target. Therefore, if 88.9 Mt GHGs are emitted in 2030, 30.3 Mt GHG removal is required. This target will be met through reducing current pollutants in international clean strategy markets [8].

In New Zealand, the government aims for 90% of electricity production to be from renewable sources by 2025 [9] and 100% by 2035 [10]. Currently, 11,349 km of transmission lines distribute electricity from remote areas, where generators are located, all over New Zealand. New Zealand consumes about 38,800 gigawatt-hours (GWh) of electricity per year. In 2017, renewable sources (hydro, geothermal, wind and solar) supplied 59%, 17%, 5% and 0.2% of the country’s electricity needs, respectively, and thermal sources (coal, diesel and gas) supplied the other 18.8% [10].

The conversion of the kinetic energy of wind into electricity via wind turbines represents wind energy. It is one of the fastest-growing renewables, as wind turbines are being installed worldwide [11]. Wind power systems are a cost-effective technology for harnessing renewable energy and have expanded annually at a dramatic rate of 25–35% globally over the last decade [12,13].

Historically, wind directions are determined from eolian sandstones [14]. In modern wind turbines, control mechanisms and operation of yaw systems maximise wind power extraction from different wind directions [15]. Accurate predictions on the wind power to be produced by the turbines are required for the grid operators from very short time scales to a few days ahead, in order to strike the balance between power supply and demand. Uncertainties and frequent fluctuations in wind speed and direction may create problems for customers who are in need of electricity supply. Further, the temporal, geographical and climatic factors, such as the particular time of the day, terrain, humidity and temperature, also affect the wind power generation that depends on generator hours of wind turbines, speed and direction of the wind, air density and position of the turbines. If the wind speed in different directions could be precisely forecast, it could bring about multiple benefits in adjusting control systems, maintenance scheduling, reducing economic and technical risks and profit maximization of the power trade. 

Due to the importance attached to the wind speed among all numerical weather prediction data, the objective of this research was focused on studying the variation in wind power generated by different directions. This is essential to predicate in order to maximise power generation from wind. Therefore, this paper presents a novel research study based on the climate model to forecast its wind power generation in different wind patterns. In this sense, this study provides an insight into an optimized off-shore site near Stewart Island to propose wind power as an alternative source for their demand.

To do this, Global Wind Atlas [16] is used to plot the wind rose in current wind patterns. In the next step, wind speed data from the Foveaux site are imported from the NASA database [17] to WRPLOT view software [18] and Homer Pro [19] to find wind frequency distribution and output power in each site.

From a technical point of view, the contributions of the paper are as follows:

1. Explore the use of wind energy for off-grid design;
2. Explore climate model for precise forecast of wind generation;
3. Explore the use of optimization software and climate models at the same time to increase power generation;
4. Define a method that could be used to optimize wind generation using climate models.

Homer Pro and WRPLOT view software are used for the integration of energy sources and analysing the resource potential. The HOMER Pro^®^ microgrid software is used to optimize the off-grid and on-grid designs in both engineering and economic aspects for residential, commercial and community purposes [19]. The WRPLOT view software is used to provide the available classes of turbine speeds for a given location [18].

The paper is organized as follows: after the Section 1, Section 2 presents the status of wind energy, Section 3 presents the methods used to evaluate the wind directions and the models used, Section 4 presents the Results and, finally, the Discussion and Conclusions are given in Section 5.

What is novel about the project described in this section is that it investigates the technical feasibility of wind generation directional wise. The results will enable us to propose the directions with maximum power generation.

## 2. The Status of Wind Energy

A literature review by Martínez et al. [20] found studies on electricity production from wind energy account for 3730 and of these, 1736 focus on off-shore deployments. Off-shore and on-shore wind is an increasingly efficient and price-competitive renewable energy source, contributing 16% of electricity produced globally by renewable energy sources in 2016 [21]. It is estimated that on-shore and off-shore wind power will generate more than a third of the total electricity needed in the medium term, becoming the primary generation source by 2050 [22]. In 2015, only 5% of New Zealand’s electricity generation was supplied from wind [23], which is expected to be 20% of NZ generation by 2035 [24].

Compared with on-shore wind energy resources, off-shore wind fields have many advantages, such as persistent wind, faster-flowing speed, higher uniformity and longer available time per year, flat sea surface and low turbulence intensity, which promotes the vigorous development of the off-shore wind power industry [25,26]. More importantly, installing wind turbines in the ocean can protect the environment [27] and save land resources [28]. The most significant environmental impacts of turbines are noise impacts, ecological effects (birds are a key issue) and visual amenities [29]. The vast ocean area provides good conditions for developing large-scale wind farms and turbines [30]. The power generation by the identical turbines in the off-shore area is 50–100% higher than in the on-shore area [31]. In more recent studies, the advantages of off-shore wind generation against on-shore wind generation have been highlighted through Life Cycle Analysis (LCA), showing a 48% improvement in the project’s sustainability [32]. Off-shore wind farms have increasingly attracted massive investment since 2015 [33]. However, the cost of electricity using off-shore wind is still high [34].

**Figure 1:**
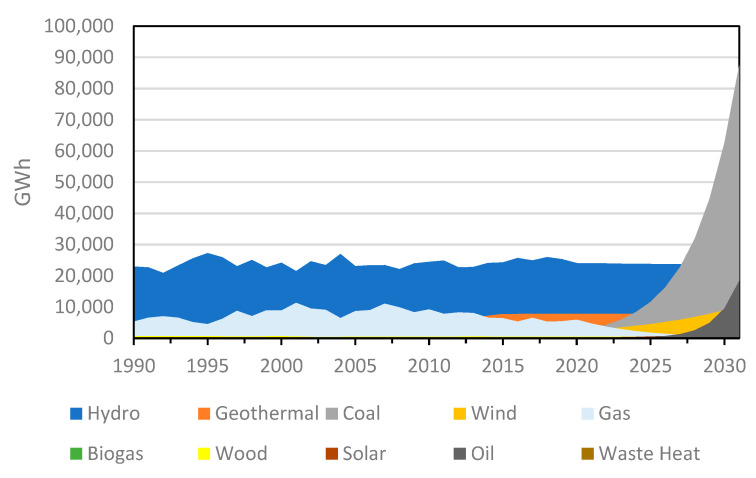
Forecast of electricity generation by fuel in New Zealand.

Historically, New Zealand’s primary renewable energy sources have been geothermal and hydropower. However, in the future, most incremental growth in renewable energy is forecasted to come from wind energy and geothermal energy capacity additions, which can be seen in the electricity generation projection chart shown in Figure 1 [35].

Wind provided 2232 GWh, or 5.1 per cent, of total electricity supply in 2019, which was 37 per cent of the capacity factor (capacity factor for wind is the amount of electricity generated in relation to the maximum output capable of being generated by installed wind turbines over a period of time assuming no downtime for maintenance) [36].

By 2030, it is predicted that wind generation in New Zealand will increase six-fold, producing as much as 20% of all electricity. Wind farm capacity can be built, as it is essentially the most cost-effective form of electricity generation, being predictable and dependable and the costs of fuel are known (wind is free) [35]. A Ministry of Business, Innovation and Employment (MBIE) report issued on June 2020 describes the desired wind generation growth stack on a decadal basis between 2020 and 2060, as tabulated in Table 1.

It shows that 2500 MW of new wind generation is required between 2020 and 2030, 1500 MW between 2030 and 2040, 2000 MW between 2040 and 2050 and 2000 MW between 2050 and 2060. To put this in context, there is currently only 690 MW of wind generation in NZ and most of this total has taken the previous 20 years to develop and construct [37].

Most existing wind generation is located in Waikato, Manawatu, Wellington and Southland. Future growth areas include Northland, west coast Waikato, South Taranaki, Hawkes Bay, Canterbury, Otago and Southland [38].

## 3. Materials and Methods

NIWA recording sites summarize every hourly measurement, illustrating patterns of turbulence and calm in different places in New Zealand. According to it, wind patterns of New Zealand are (a) Christchurch: gentle winds from most directions, (b) Bluff: strong westerlies, moderate easterlies and southerlies, (c) Dunedin: wind from most directions, except the north-east and south, (d) Greymouth: strong south-westerlies and easterlies, (e) Blenheim: moderate westerlies and north westerlies, (f) Auckland: dominated by westerlies, south westerlies and north easterlies, (g) Awakino: strong westerlies and moderate south-easterlies, (h) Gisborne: a calm year of mostly gentle winds, (i) Wellington: strong northerlies and southerlies and (j) Akitio: wind from all directions, with especially strong north-westerlies [39]. Stewart Island is selected as an off-grid site to evaluate the maximum possibility of wind power generation as an alternative to the current diesel station. Then, to find Foveaux as an optimized site for wind power generation, NIWA and MetOcean models were used. Global Wind Atlas [16] and WRPLOT view software [18] were used to identify wind rose and distribution of wind speeds in different ranges. Next, to find accurate wind power in different directions, data from SIESA [40] were analysed according to cut-in speed of selected turbines. All steps for identifying the maximum wind power generation from Stewart Island will be explained with details in the next sub-sections, which are:

- Selecting a suitable site for the case study.
- Obtaining environmental data for the energy resources or wind speeds.
- Turbine selection.

### 3.1. Site Selection

**Figure 2:**
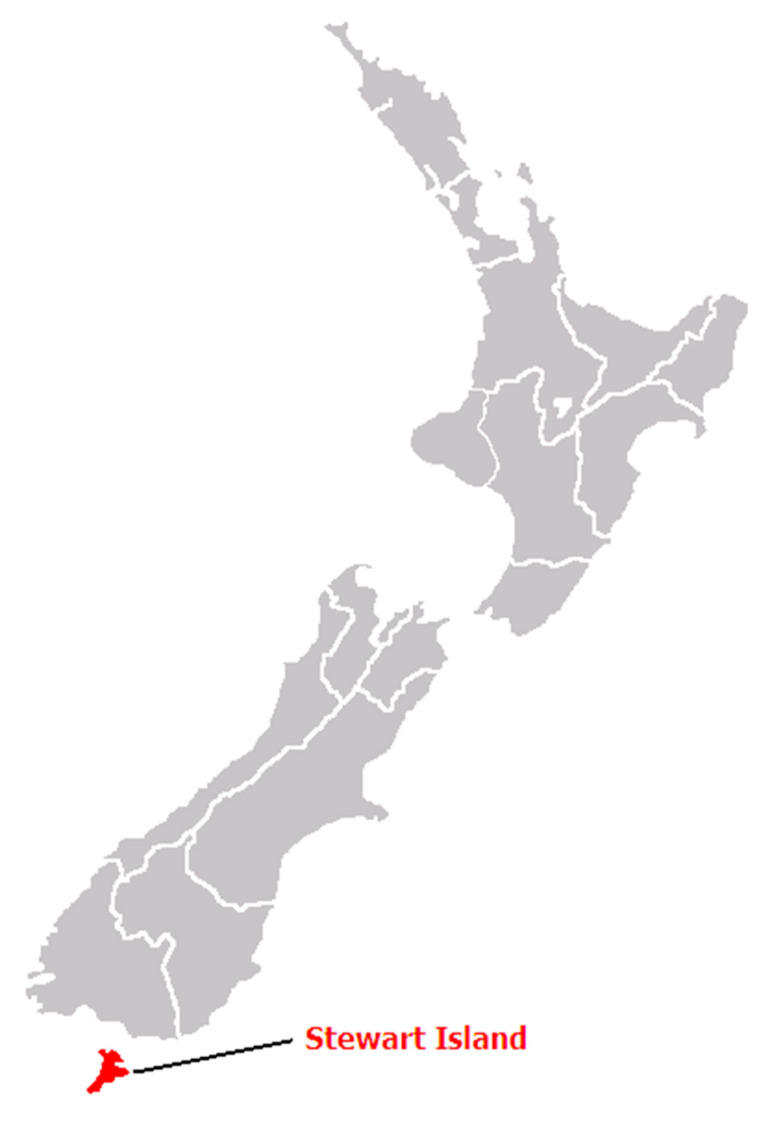
Location of Stewart Island.

Stewart Island is selected as a case study to evaluate wind power generation for power supply. The island is a remote coastal community isolated from a national supply grid (Figure 2). The cost of electricity is much higher than on the grid-connected mainland because power is supplied to just 408 customers from a small diesel power station located centrally in the island’s main settlement Oban by the Stewart Island Electrical Supply Authority (SIESA). Its retail charge is 62 c/kWh, of which 23 c/kWh is the direct cost of operating five diesel generators. Local residents believe that reducing the consumption of diesel and having a renewable source of electricity generation are two of the island’s highest priorities. Therefore, replacing diesel as a renewable energy source is a top priority for residents [40].

### 3.2. Obtaining Environmental Data

**Figure 3:**
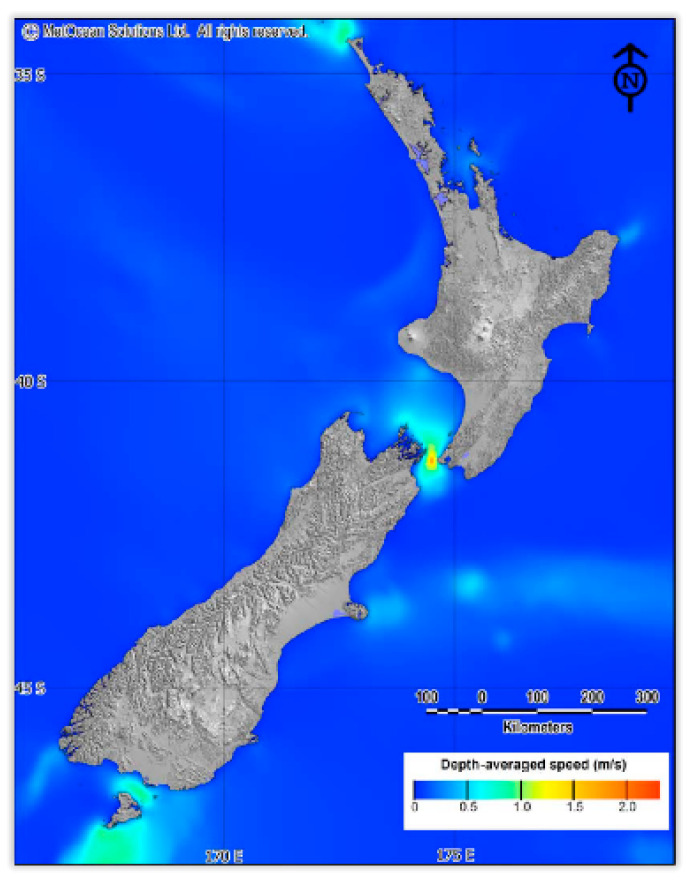
National depth-averaged tidal current speeds for mean spring flows (in m/s) [42].

Tidal current data were obtained from a simulation model, which MetOcean Solutions Limited (MSL) conducted on an NZ-wide grid with a 0.06° resolution (5.6 × 6.6 km). The simulation nested high-resolution domains over Foveaux Strait (0.004°; 340 × 450 m), shown in Figure 3. The Princeton Ocean Model (POM) was used to hindcast the tidal current in a vertically integrated two-dimensional mode with boundaries provided by the global TPX07.1 solution. According to the MetOcean atmospheric model, the tidal currents in four areas, (i) Cape Regina (in the north), (ii) Cook Strait (between two Islands), (iii) Foveaux Strait (in the north of Stewart Island) and (iv) south of Stewart Island, are more than 1 m/second. Cape Reinga and the south of Stewart Island were not considered further, since both areas are significant distances from existing electricity infrastructure and potential markets, both of which would considerably increase the cost of potential projects in these areas [41]. Therefore, two areas are suitable (i) Cook Strait in the south of the northern island and (ii) the Foveaux Strait in the south of the southern island in Stewart Island.

**Figure 4:**
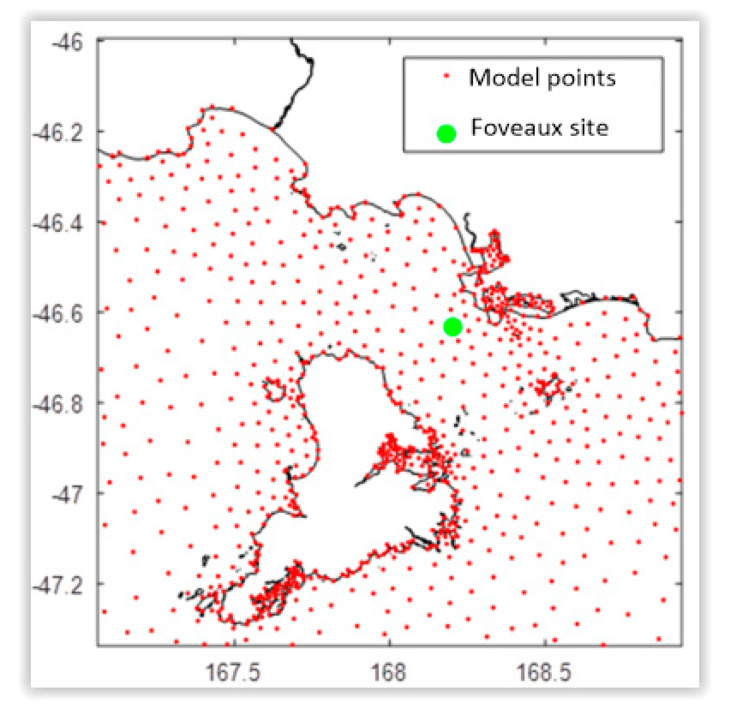
Tidal current model points near Stewart Island from NIWA.

**Figure 5:**
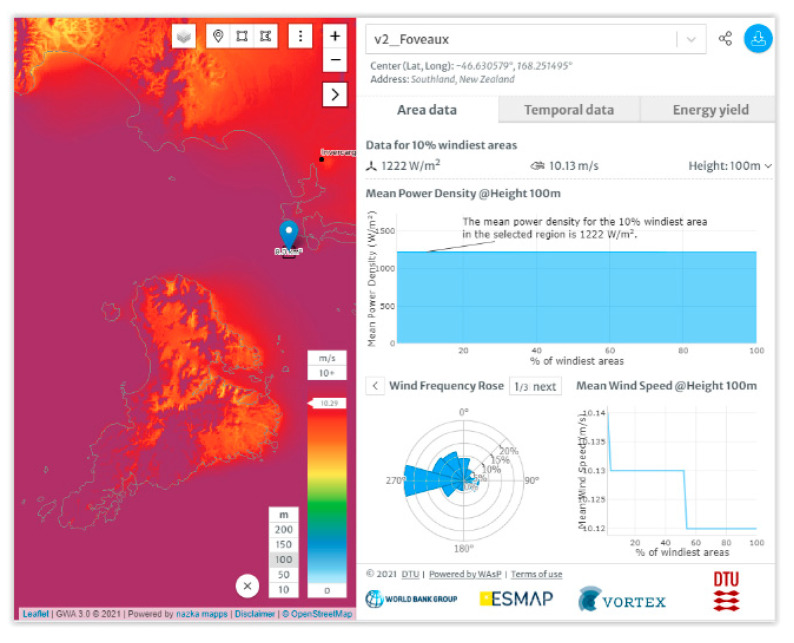
Wind conditions near Stewart Island (**left**) and at the Foveaux site (**right**) [16].

One of these points, referred to as the Foveaux site (Figure 4), was selected for the case study because, despite being on the opposite side of Foveaux Strait, it appears to have stronger currents (better generation potential) than points close to Stewart Island’s coast and also because the Global Wind Atlas model [16] indicates that this point is in an area with frequent strong winds (high potential for wind energy), as shown in Figure 5. For New Zealand, Global Wind Atlas extrapolates data from a network of meteorologic recorder stations operated by the New Zealand Meteorological Service (NZMS) and the National Institute of Water and Atmosphere (NIWA).

**Figure 6:**
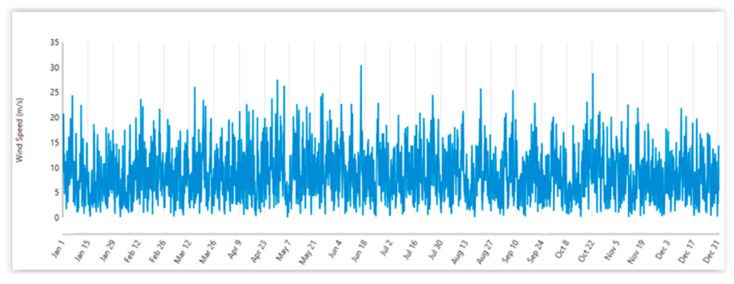
Annual wind speed for Foveaux site.

The environmental parameters of the site are summarised in Table 2 and Figure 6.

The wind speed values for Foveaux can be automatically downloaded, from which the annual average wind speed can be calculated using Homer Pro, as shown in Figure 6.

### 3.3. Turbine Selection

Given the infrastructure on Stewart Island, it is considered that smaller-scale wind turbines would be more suitable for installation on the island. The capacity rate of a selected turbine needs to be aligned with the current fluctuation load, which is between 106 and 209 kW [43]. A suitable wind turbine in terms of low cut-in speed, a generating capacity capable of meeting peak load, supplying a substantial percentage of peak load and low purchase, installation and maintenance costs is the XANT M-21 (100 kW) [43]. The pertinent details for the XANT wind turbine are given in Table 3.

XANT is based in Brussels, Belgium, and commenced operations in 2011. As of August 2018, they had installed ten turbines, including two in Scotland and two in Alaska. Other units are installed in Singapore and Vanuatu [40]. It has an ice-detection sensor, which prevents ice accumulation that causes a reduction in energy yield and safety of the XANT turbine [44].

**Figure 7:**
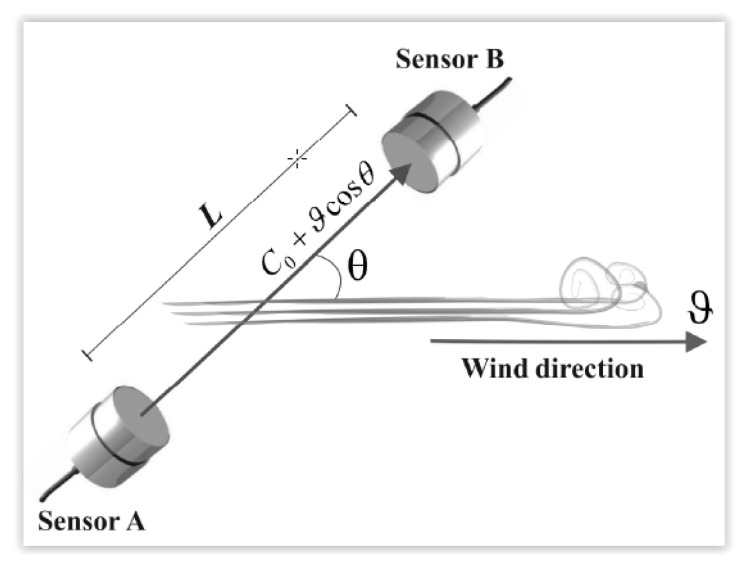
Wind speed measurement sensor configuration.

A typical configuration to measure the wind speed using ultrasonic sensors consists of aligning sensors with a specific angle to the direction of the wind, as shown in Figure 7 [45].

The wind speed for the configuration can be calculated as:

(1) $$\vartheta =\frac{1}{\cos\theta }\left(\frac{L}{T_{0}F}-C_{0}\right)$$

where: ϑ is the wind speed, T_0_F is the time of flight needed for the ultrasonic wave to travel from the transmitting to the receiving sensor (sensor B), L is the distance between the sensors, θ is the angle between the wind propagation and sensor lining directions and C_0_ is the sound speed in the steady air, which varies mainly with the temperature and can be determined by measuring the temperature T_K_ = T + 273.15 (T_k_ in degrees Kelvin and T in degrees Celsius) [46], i.e.,

(2) $$C_{0}=20.074\sqrt{T_{K}}$$

**Figure 8:**
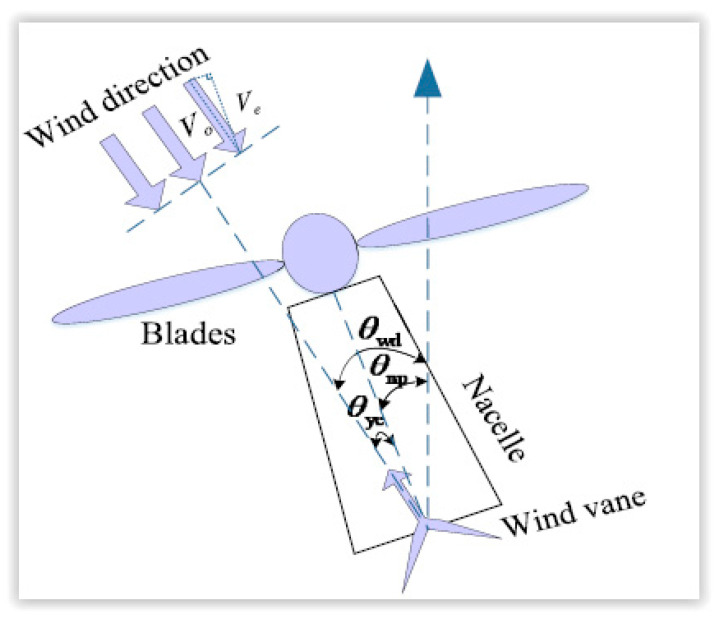
Schematic diagram of wind direction measurement [47].

In wind turbines, the yaw system is used to rotate the face of the turbine to the wind. Wind direction sensors are generally installed at the rear of the nacelle, as shown in Figure 8 [47].

## 4. Results

**Figure 9:**
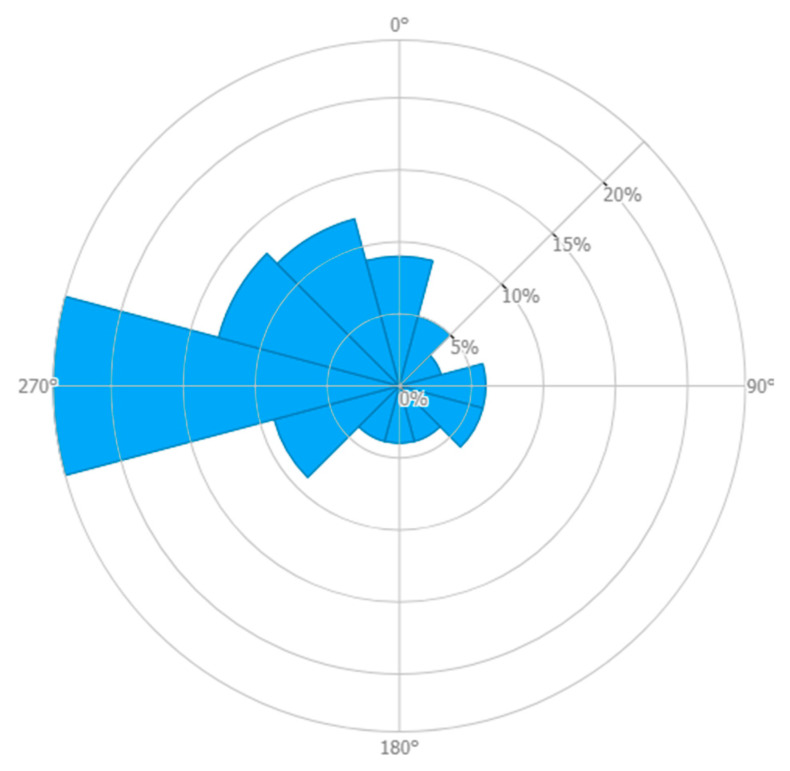
Wind rose of Foveaux [16].

Global Wind Atlas was used to find wind rose of Foveaux, as shown in Figure 9 [16].

The directions used in this section are as below, where N, S, W and E refer to north, south, west and east, respectively. They are described in more detail in Table 4.

**Figure 10:**
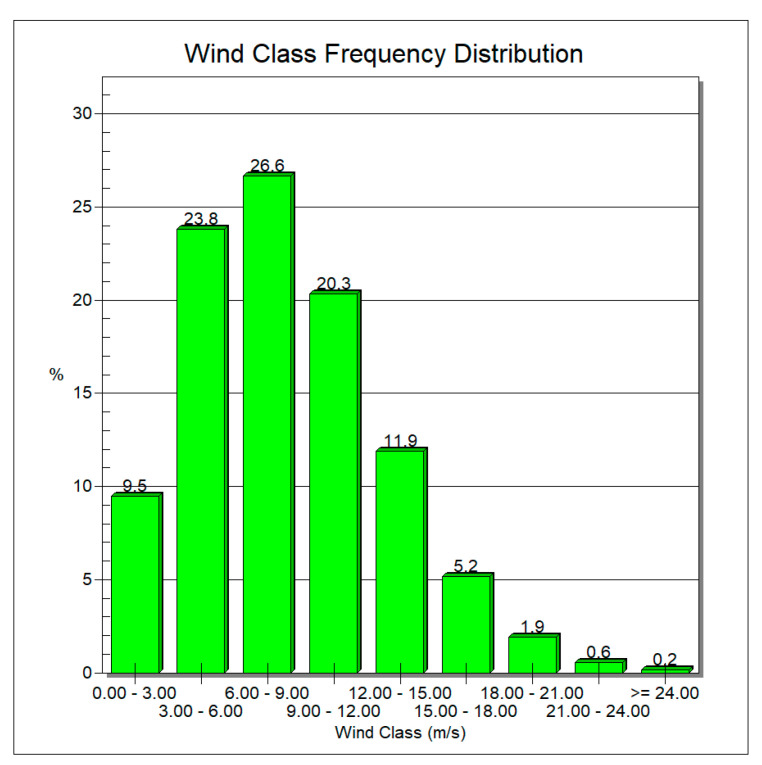
Percentage of wind blowing in different wind speed ranges at the Foveaux site.

It can be seen that the dominant direction for Foveaux is west. WRPLOT view software [18] was used to plot wind frequency distribution, downloaded from the NASA Surface Meteorology and Solar Energy database [17] at the Foveaux site. As seen in Figure 10, for 90.5% of the year, the wind speed is more than the cut-in speed, so the XANT M-21 wind turbine will be suitable for electricity generation at the Foveaux site.

**Figure 11:**
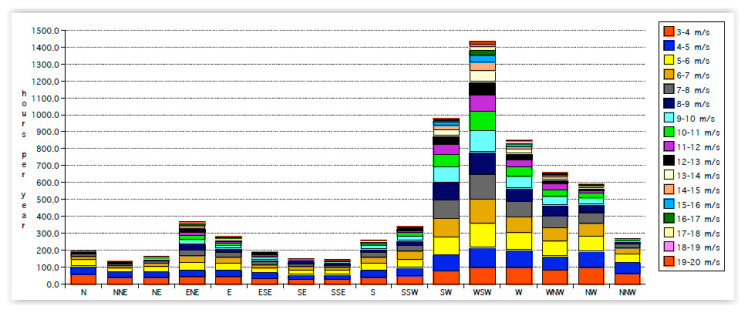
The yearly hour’s wind blows from different directions at different speeds.

The yearly hour wind blows from different directions on Stewart Island are shown in Figure 11. It is generated from a wind model commissioned by Stewart Island Electricity Supply (SIESA) [40]. The wind model adopts local Stewart Island meteorological records according to terrain elevation and exposure.

**Figure 12:**
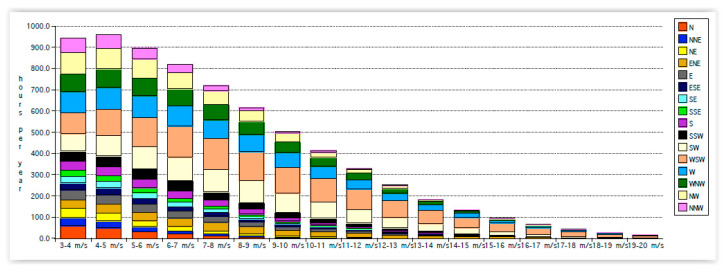
Wind directions for different wind speeds.

Figure 11 can be replotted, showing wind directions in different wind speeds, as shown in Figure 12.

**Figure 13:**
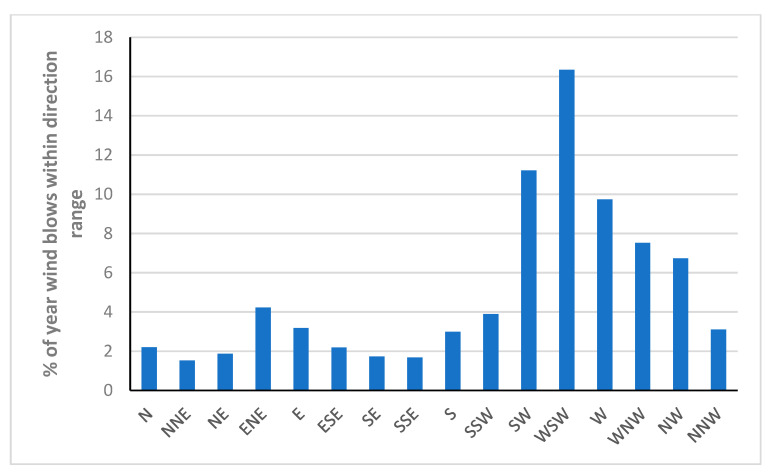
The percentage of year when the wind blows within direction ranges.

Figure 13 shows the years when the wind blows within direction ranges. The maximum percentage can be seen in WSW (%16.3), SW (%11.2) and W (%9.7) directions, respectively.

**Figure 14:**
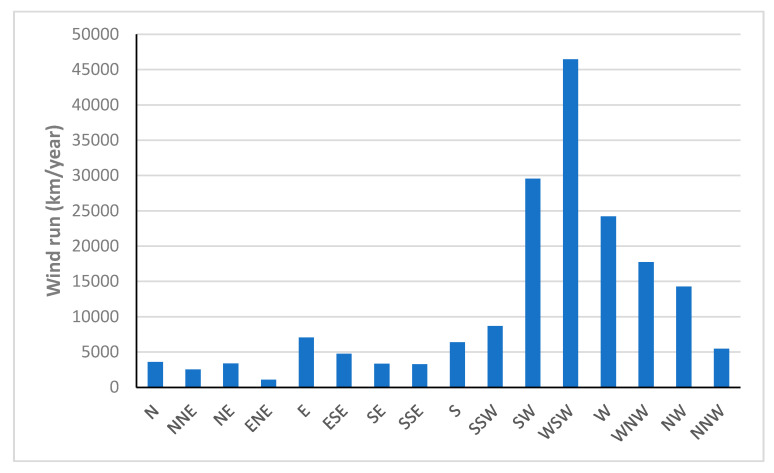
The annual wind run in different directions.

Figure 14 shows the annual wind run from different directions. The maximum wind run can be seen in WSW (46,477 km/year), SW (29,546 km/year) and W (24,216 km/year) directions, respectively.

**Figure 15:**
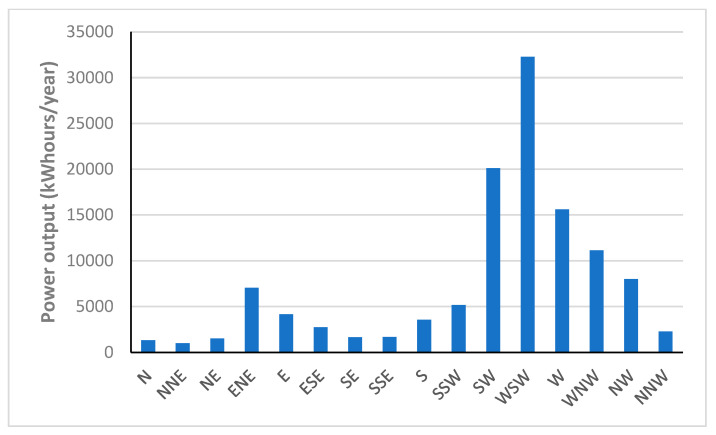
The annual wind power from different directions.

Figure 15 shows the annual power output the turbine generates from different directions. The maximum wind run can be seen in WSW (32,299 kW hours/year), SW (20,111 kW hours/year) and W (15,622 kW hours/year) directions, respectively.

Therefore, maximum wind power can be generated in WSW and SW directions.

## 5. Discussion and Conclusions

This paper investigated climate models for predicting generated wind power in different wind patterns for Stewart Island to maximise power generation in a remote off-grid coastal community and avoid the detrimental effects of off-grid diesel generation on the environment.

Stewart Island was selected as a case study because it is typical of isolated coastal communities in New Zealand, which depend on small locally operated diesel power stations or single-household petrol generators. Based on the selection of turbine and Foveaux as facility sites and by including the resource data of the wind speed, the design provides the capacity of wind energy from different directions. It is generated from a wind model commissioned by Stewart Island Electricity Supply (SIESA).

Applying control mechanisms and the operation of yaw systems maximise wind power extraction from different wind directions. To do this, NIWA and MetOcean models were used to find Foveaux as an optimised site north of Stewart Island. Then, Global Wind Atlas was used to plot wind rose of current wind patterns in Foveaux. It indicates that the dominant direction for Foveaux is west. WRPLOT view software was used to plot wind frequency distribution downloaded from NASA Surface Meteorology and Solar Energy database at the Foveaux site. Using the XANT M-21 wind turbine provides electricity for 90.5% of the year for Oban.

An industry-recognised simulation software package, Homer Pro, was used to import wind speeds from the NASA Surface Meteorology and Solar Energy database at the Foveaux site.

The results, as presented and discussed in Section 3, enable several conclusions:

▪ There is enough wind to generate electricity for 90% of the year at the Foveaux site. However, this site is on the other side of Foveaux Strait, 40 km away from Stewart Island´s community at Oban.
▪ DC electricity from the off-shore Foveaux site can be integrated with AC electricity from the existing on-shore diesel generators at Oban. The design solution is to feed power through a DC marine cable to a DC-AC converter at the Stewart Island shore, then through an AC land cable to a system controller at Oban´s diesel generator station.
▪ The design can be optimised in a way that enables Stewart Island´s power to be supplied partly from renewable sources. Simulation of various climate models shows several directions, which look promising. The maximum wind power can be generated in WSW and SW directions.
▪ The investigation is worth implementing because it could supply Stewart Island´s power demand from a renewable source, reducing the detrimental effects of using diesel fuel on the environment.
▪ It is unknown whether the design can be constructed at an affordable cost because the Homer Pro financial analysis module does not include construction or installation. Further, this research just focused on climate models.
▪ A cost–benefit analysis is needed to determine whether the supply authority can recover all construction, installation, operating and maintenance/replacement costs from a lower charge to the community than its present retail charge.

## Figures and Tables

**Table 1:** Minimum cumulative MW of new wind generation until 2060 [37].

Year	2030	2040	2050	2060
**MW (Cumulative)**	2500	4000	6000	8000

**Table 2:** The environmental parameters of the Foveaux site.

Location	Latitude (deg)	Longitude (deg)	Annual Average Wind Speed (m/s)
Foveaux	−46.6325° S	168.2025° E	8.31

**Table 3:** Wind turbine details [19].

Wind Turbine	Value
Name	XANT M-21 [100 kW]
Rated Capacity (kW)	100
Manufacturer	XANT
Cut-in wind speed	3 m/s
Cut-out wind speed	20 m/s
Rated wind speed	11 m/s
Hub height	31.8 m
Swept area	346.36 m^2^
Rotor diameter	21 m

**Table 4:** Directions of wind in degree.

Direction	Degree
N	349–11
NNE	12–33
NE	34–56
ENE	57–78
E	79–101
ESE	102–123
SE	124–146
SSE	147–168
S	169–191
SSW	192–213
SW	214–236
WSW	237–258
W	259–281
WNW	282–303
NW	304–326
NNW	327–348

## Data Availability

Not applicable.
